# Novel, one-step synthesis of zwitterionic polymer nanoparticles via distillation-precipitation polymerization and its application for dye removal membrane

**DOI:** 10.1038/s41598-017-16131-9

**Published:** 2017-11-21

**Authors:** G. P. Syed Ibrahim, Arun M. Isloor, Inamuddin, Abdullah M. Asiri, Norafiqah Ismail, Ahmed Fauzi Ismail, Ghulam Md Ashraf

**Affiliations:** 1 Membrane Technology Laboratory, Department of Chemistry, National Institute of Technology Karnataka, Surathkal, Mangalore, 575025 India; 2 Chemistry Department, Faculty of Science, King Abdulaziz University, Jeddah, 21589 Saudi Arabia; 3 Centre of Excellence for Advanced Materials Research, King Abdulaziz University, Jeddah, 21589 Saudi Arabia; 4 Advanced Functional Materials Laboratory, Department of Applied Chemistry, Faculty of Engineering and Technology, Aligarh Muslim University, Aligarh, 202002 India; 5 Advanced Membrane Technology Research Center (AMTEC), Universiti Teknologi Malaysia, 81310 Skudai, Johor Bahru Malaysia; 6 King Fahd Medical Research Center, King Abdulaziz University, Jeddah, 21589 Saudi Arabia

## Abstract

In this work, poly(MBAAm-co-SBMA) zwitterionic polymer nanoparticles were synthesized in one-step via distillation-precipitation polymerization (DPP) and were characterized. [2-(methacryloyloxy)ethyl]dimethyl-(3-sulfopropyl)ammonium hydroxide (SBMA) as monomer and N, N′-methylene bis(acrylamide) (MBAAm) as cross-linker are used for the synthesis of nanoparticles. As  far as our knowledge, this is the first such report on the synthesis of poly(MBAAm-co-SBMA) nanoparticles via DPP. The newly synthesized nanoparticles were further employed for the surface modification of polysulfone (PSF) hollow fiber membranes for dye removal. The modified hollow fiber membrane exhibited the improved permeability (56 L/ m^2^ h bar) and dye removal (>98% of Reactive Black 5 and >80.7% of Reactive orange 16) with the high permeation of salts. Therefore, the as-prepared membrane can have potential application in textile and industrial wastewater treatment.

## Introduction

In the recent years, the discharge of colored micropollutants into the water stream has elevated widespread concernas dyes are toxic, non-biodegradable and carcinogenic^1–3^. Anionic dyes are recognized as contaminants in wastewater, which are broadly employed in industries like paper, textile, and plastics^4^. The dyes can be categorized into three types, viz. azo, anthraquinone and triphenylmethane. Reactive Black 5 and reactive orange 16 are falling below the category of azo dyes. These acid dyes are used for coloring the cellulose-based fabrics such as cotton. Since reactive dyes are accompanying with moderate rates of fixation, dyeing with reactive dyes always associated with serious environmental problems^5^. The complex structure of the acid dyes makes it insensitive to biodegradation and chemical oxidation. Consequently, it produces secondary pollutants during oxidation^6,7^. With the intention of solving this environmental pollution, it is critical to eliminate dyes from effluent before discharging. A number of methods such as flocculation, adsorption, photodegradation and chemical oxidation are available for the treatment of wastewater^8–12^. However, these methods are not cost-effective, less energy efficient produces solid wastes and so on^13^. Therefore, a new method for treating this wastewater is extremely needed. Membrane separation techniques have been proved to be the potential alternative^14–18^ to remove dye from the wastewater. In addition, it has many advantages like energy efficient, low-cost, non-toxic, easy to scale up, comprising no chemical reaction, high efficiency and produces less solid waste^19^. In general, rejection of these low molecular weight dye molecules are carried out using nanofiltration (NF) and reverse osmosis (RO) membranes^20–23^. Nevertheless, these separation processes are suffering from some downsides such low flux and high cost^24–27^. Ultrafiltration (UF) is one of the emerging pretreatment technology for the RO and NF^28^. Specifically, hollow fiber UF membranes are dominating over the flat sheet due to their increased surface area per unit of module volume^29,30^. In addition, UF membranes are talented to remove suspended solids, bacteria and high molecular weight solute from water^31^.

Polysulfone (PSF) is one amongst the versatile polymeric material for the preparation of hollow fiber (HF) membranes. It has very high thermal, mechanical and chemical resistance along with outstanding film forming ability^32,33^. The other polymeric materials such polyetherimide (PEI) undergoes hydrolysis under basic condition^34^, chitosan (CS) which is insoluble in organic solvents^35^ and polyphenylsulfone (PPSU) is brittle in nature^36^. Therefore PSF is superior to other polymeric materials. Nonetheless, PSF membrane is vulnerable to severe fouling of very short duration. The fouling is caused by the less hydrophilic nature of the PSF material. Consequently, the foulant forms a cake-like layer, which reduces the permeation rate of the water as well as increase the hydrophobicity and operational cost^37,38^. Mauter *et al*. reported the effect of adding PEI modified silver nanoparticle into PSF UF membranes. The results indicated that surface modified PSF membranes exhibited increased antifouling and antimicrobial activity^39^. Fan *et al*. explored the antifouling and hydrophilicity of the PANI/PSF nanocomposite membranes. The nanocomposite membrane demonstrated enhanced hydrophilicity and antifouling nature, as a result the nanocomposite membrane exhibited high permeability without losing its rejection performance^40^. Joseph *et al*. reviewed that incorporation of zwitterionic thin or thick film on the surface reduced the protein adsorption^41^. Tao *et al*. improved the blood compatibility of PSF membrane by the chemical modification of PSF with zwitterionic polymer brush. The results also indicated that the introduction of the zwitterionic functional group increased the surface hydrophilicity^42^. Haijun *et al*. investigated the effect of grafting of the zwitterionic molecule on PSF UF membrane. The results showed that surface hydrophilicity and antifouling nature enhances while increasing the grafting time^43^. In current years, it has been reported that incorporation of zwitterionic nanoparticles exhibited improved hydrophilicity, permeability, and antifouling performances^44–47^. The zwitterionic material has ample ionic groups which provide strong electrostatic interaction with water molecules, therefore it provides stronger and denser hydration layer over the membrane surface^48^. In addition, the polymer matrix is well miscible with hydrophobic chains of the zwitterionic polymers. Gang *et al*. employed zwitterionic polymer brush on TFC membrane to bestow anti-biofouling activity^49^. Liu *et al*. investigated the effect of adding zwitterionic-CNT for the preparation of ion selective membrane. The added nanomaterial enhanced the mono/multivalent ion selectivity when compared to the pristine CNT nanocomposite membrane^50^.

Among the polymerization processes, distillation-precipitation polymerization (DPP) is the facile process and recently developed by Feng *et al*.^51^. It is a unique method to prepare nanoparticles with uniform size and shape without adding any surfactant or stabilizer^52,53^. Additionally, this process can be scaled up since the refluxing solvent can bestow effective mixing and oxygen-free environment^54^. In comparison with the classical polymerization processes such as atom-transfer radical-polymerization (ATRP), group transfer polymerization (GTP), catalytic chain transfer polymerization and radical polymerization, DPP holds superior advantages like lesser reaction time (typically 2–3 h), cheap starting materials, no metal catalyst, and ligand are required, no sophisticated apparatus required, reaction at atmospheric condition, atom economy and easy isolation method. The mechanism of DPP follows the order of radical initiation of monomer or cross-linker and subsequent chain propagation by chain addition, which results in precipitation of polymeric nanomaterial. The increased colloidal stability of the prepared nanoparticles could be attributed to the surface charge, which is affecting through electrostatic repulsion. Thus, the aggregation of the nanoparticles was circumvented. According to Feng *et al*. the nanoparticle size increases with the increase of monomer and initiator concentration^51^. The increased concentration of cross-linking agent such as MBAAm increases the hydrophilicity of the material^55^. Among the solvents, ACN was chosen as the reaction solvent, however, protic solvents such as ethanol or methanol forms aggregate through hydrogen bond formation^56^.

In the present study, poly(MBAAm-co-SBMA) zwitterionic polymer nanoparticles were synthesized by SBMA as monomer and MBAA as cross-linking agent via distillation-precipitation polymerization (DPP). The as-synthesized nanoparticles were characterized by FT-IR, TEM, SEM, BET, TGA, XRD and zeta potential analysis. The PSF HF membranes were prepared with the different amount of nanoparticles by dry/wet phase inversion method. Moreover, SEM, contact angle, porosity, water uptake, zeta potential, pure water permeability and antifouling study characterized the as-made PSF HF membranes. Furthermore, the nanocomposite membrane explored for the dyes such as reactive black 5 (RB 5) and reactive orange 16 (RO 16) rejection.

## Materials and Methods

### Materials

Polysulfone (PSF, P-1700) was purchased in the form of pellets from Solvay Specialty Polymers (China). The solvents N-methyl pyrrolidone (NMP) and acetonitrile (ACN) were obtained from Merck. Polyvinylpyrrolidone (PVP K-30), bovine serum albumin (BSA), [2-(methacryloyloxy)ethyl]dimethyl-(3-sulfopropyl)ammonium hydroxide (SBMA), N,N′-methylene bis(acrylamide) (MBAAm), reactive black 5 (RB 5), reactive orange 16 (RO 16) and azobisisobutyronitrile (AIBN) were procured from Sigma-Aldrich.

### Synthesis of poly(MBAAm-co-SBMA) nanoparticles

**Figure 1:**
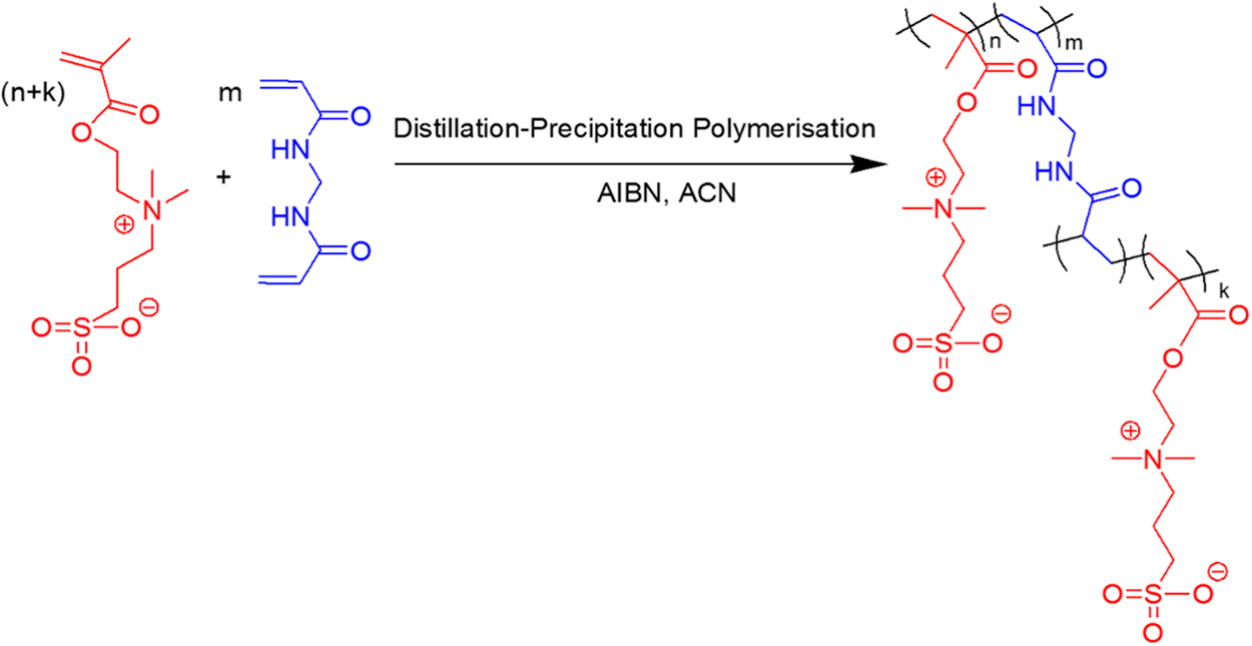
Synthetic route to poly(MBAAm-co-SBMA).

In a typical DPP process, SBMA (0.2 g, 0.71 mmol), MBAAm (1.0 g, 6.4 mmol), AIBN (0.0225 g, 0.13 mmol), ACN (100 mL) were taken in a 250 mL single neck round bottom flask (RBF), purged with N_2_ for 30 min to remove the dissolved oxygen. The RBF containing reaction mass (RM) was connected to the Dean-Stark receiver. The RM was heated to 75 °C for 10 min. The temperature of the oil bath was slowly increased to 100 °C to keep the reaction proceeding under reflux. About 35 mL of ACN was distilled out from the RM through Dean-Stark receiver over 1 h. Then the RM was cooled to room temperature and stirred for 1 h. The nanoparticles were filtered and washed with (2 × 20 mL) ACN to remove the unreacted monomer and oligomer. The nanoparticles were dried under vacuum (−25 Hg) at 50 °C for 12 h to yield 1.12 g of white powder. The synthetic route of nanoparticles is represented in Figure 1.

### Hollow fiber (HF) membrane preparation

**Figure 2:**
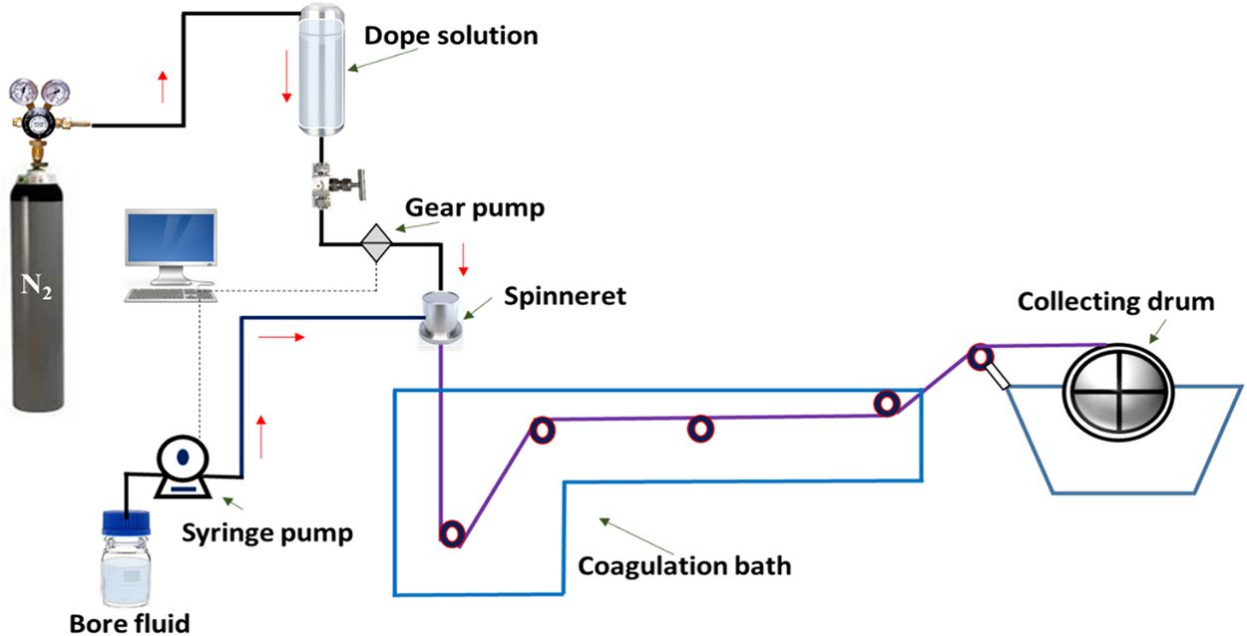
An illustration scheme of HF membrane preparation.

The PSF/poly(MBAAm-co-SBMA) HF membranes prepared by dry/wet phase inversion method^57^. The dope solution compositions are depicted in Table 1 and spinning parameters are tabulated in Table 2. For the preparation of M-3, 0.1 g of poly(MBAAm-co-SBMA) nanoparticles were dispersed in 79 g of NMP by sonicating (40 kHz, 60 W Spectralab) for 30 min. Added 20 g of PSF and 1 g of PVP as a pore-forming agent to the dope solution and stirred at 60 °C for 12 h. The dope solution was degassed for 30 min using sonication. The HF membranes were spun by keeping the bore and dope extrusion rate constant. The extruded HF membrane underwent phase inversion in the coagulation bath. The as-made HF membranes were immersed in distilled water for 24 h by changing the water periodically. The membranes were retained in 20 wt% glycerol in water for further 24 h to avoid the pore shrinkage. The post-treated membranes were dried at room temperature for future usage. The illustration scheme of HF membrane preparation has been given in Figure 2.

**Table 1:** The dope solution composition.

Membrane	PSF (g)	PVP (g)	NMP (g)	poly(MBAAm-co-SBMA) (g)
M-0	20	1	79	0
M-1	20	1	79	0.02
M-2	20	1	79	0.05
M-3	20	1	79	0.10
M-4	20	1	79	0.20

**Table 2:** Spinning parameters of PSF/poly(MBAAm-co-SBMA) HF membranes.

Parameters	Conditions
Spinneret (mm)	1.1/0.55 (OD/ID)
Dope extrusion rate (mL/min)	3.0
Bore flow rate (mL/min)	2.5
Bore fluid	Distilled water
Air gap (cm)	1.0
Humidity (%)	60
Coagulation bath	Tap water
Coagulation bath temperature (°C)	27.0
Collection drum speed (RPM)	7.0

### Nanoparticles and membranes characterizations

Morphology of the synthesized poly(MBAAm-co-SBMA) nanoparticles was visualized using transmission electron microscopy (TEM) (JEOL JEM-2200FS) with an accelerating voltage of 200 kV, and Field Emission scanning electron microscopy (FESEM) (HITACHI SU5000). The elemental mapping was carried out using Energy-dispersive X-ray spectroscopy (EDX) (X-act Oxford Instruments). The functional group identification was done by FT-IR (Bruker Alpha) spectrophotometer. Each sample was made into KBr pellet and analyzed at the resolution of 2 cm^−1^ with 24 scans in the range of 4000–500 cm^−1^. The thermal stability was measured by using thermogravimetric analysis (TGA) (HITA CHI EXSTAR 6300) in the temperature range of 30–800 °C at a heating rate of 10 °C min^−1^ under N_2_ atmosphere. The Bruner-Emmet-Teller (BET) surface area, pore volume and mean pore diameter were measured using Smart instruments (Smart Sorb 92/93). The surface charge and hydrodynamic diameter of the nanoparticles were measured by dispersing 5 mg of sample in distilled water (pH ~6.5) using HORIBA SZ-100 nanoparticle analyzer. The polymorphism of the poly(MBAAm-co-SBMA) nanoparticles was analyzed by benchtop powder X-ray diffractometer (XRD) (Rigaku, mini Flex 600) with Cu Kα as an X-ray source. The as-prepared membrane samples were dried at 50 °C for 12 h before the analysis. The cross-sectional images of the membranes were visualized by SEM (HITACHI TM3000). The samples were sputtered with platinum to bestow conductivity. The surface hydrophilicity of the membranes was measured using water contact angle analyzer (OCA, Dataphysics instrument) at room temperature. The zeta potential of the membrane surface was analyzed by the electrokinetic analyzer (Surpass Anton Paar) with 0.001 M KCl as the background electrolyte. The presence of nanoparticles in the membrane matrix was confirmed by X-ray photoelectron spectroscopy (XPS, THERMO FISHER Scientific K-ALPHA) analysis. Al Kα radiation (1486.6 eV) was used as an X-ray source and take-off angle was 20°_._

### Porosity and water uptake studies

The porosity and water uptake studies were carried out according to the literature^58,59^. Briefly, the membrane samples were cut into a length of 2 cm and dipped in distilled water for 24 h. The sample was taken out and water on the surface was wiped out gently with tissue paper. The wet weight of the sample was noted and dried at 60 °C until the constant weight. The dry weight of the sample noted and water uptake was calculated using the following equation.

1 $$ \% \,{\rm{Water}}\,{\rm{uptake}}=(\frac{{W}_{w}-{W}_{d}}{{W}_{w}})\times 100$$

Where ‘W_w_’ is the wet weight of the membrane and ‘W_d_’ is the dry weight of the membrane.

The percentage of porosity (ε) was calculated by using the following equation.

2 $${\rm{\varepsilon }}( \% )=\frac{{W}_{w}-{W}_{d}}{A\times l\times p}\times 100$$

Where, ‘*l*’ is the thickness of the membrane (cm), ‘A’ is the area of membrane (cm^2^) and ‘p’ is the density of pure water (0.998 g cm^−3^).

### Molecular weight cut-off (MWCO) study

The MWCO of M-3 membrane was determined by filtering a series polyethylene glycol (PEG) with average molecular weight of 2000, 4000, 6000 and 10,000 Da^60^. The rejection coefficient of 500 ppm of PEG solutions were assessed at 1 bar pressure. The solute concentration was measured in terms of total organic carbon (TOC) with TOC-L SHIMADZU TOC analyzer. The percentage of rejection was calculated using the following equation.

3 $$ \% \,{\rm{of}}\,{\rm{rejection}}=(1-\frac{{C}_{p}}{{C}_{f}})\times 100$$

Where ‘C_p_’ and ‘C_f_’ are the solute concentrations of permeate and feed respectively.

### Permeation and antifouling study

In the permeation study, 10 cm length of HF membrane sample was cut and potted using epoxy adhesive. All the experiments were carried out in the lab made cross flow apparatus. At first, the membranes were compacted for 30 min at 2 bar pressure. The pure water permeability (PWP) ‘J_w1_’ was measured using the following equation at 1 bar for 60 min.

4 $${{\rm{J}}}_{{\rm{w1}}}=\frac{Q}{nA{\rm{\Delta }}P}$$

Where ‘Q’ is the amount of water collected (L h^−1^), ‘n’ is the number of hollow fiber membrane, ‘∆P’ is the applied pressure (bar), ‘A’ is effective area of the hollow fiber membrane (m^2^) and ‘J_w1_’ is expressed in (L /m^2^ h bar).

The percentage dye rejection was calculated by the above equation (3). An aqueous solution of RB 5 and RO 16 were prepared at the concentration of 100 ppm. The solute concentration was measured using UV-Vis spectrophotometer (HACH DR 5000) at the λ_max_ of 592 nm and 494 nm for RB 5 and RO 16.

The antifouling performance of the membranes was studied by calculating the flux recovery ratio (FRR)^61^. In this study BSA (0.8 g L^−1^) was used as a model foulant. The ‘J_w1_’ was measured by calculating the clean water permeability for 40 min. The BSA solution was passed through the membrane surface for another 40 min and ‘J_p_’ was calculated. The membranes after fouling with BSA solution was washed in running tap water for 10 min and ‘J_w2_’ was measured as like ‘J_w1_’. The FRR can be calculated using the following equation.

5 $${\rm{FRR}}\,( \% )=(\frac{{J}_{w2}}{{J}_{w1}})\times 100$$

## Results and Discussion

### Characterization of poly(MBAAm-co-SBMA) nanoparticles

#### FT-IR and TEM analyses

**Figure 3:**
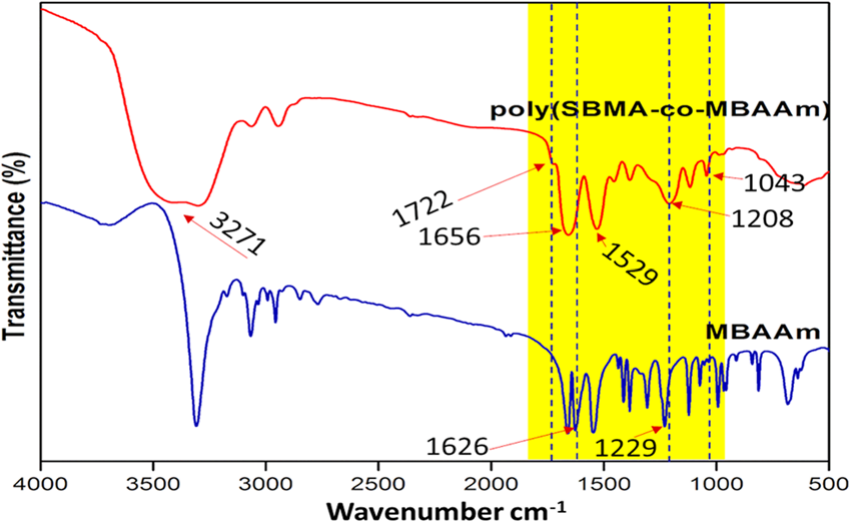
FT-IR spectra of poly(MBAAm-co-SBMA) and MBAAm.

**Figure 4:**
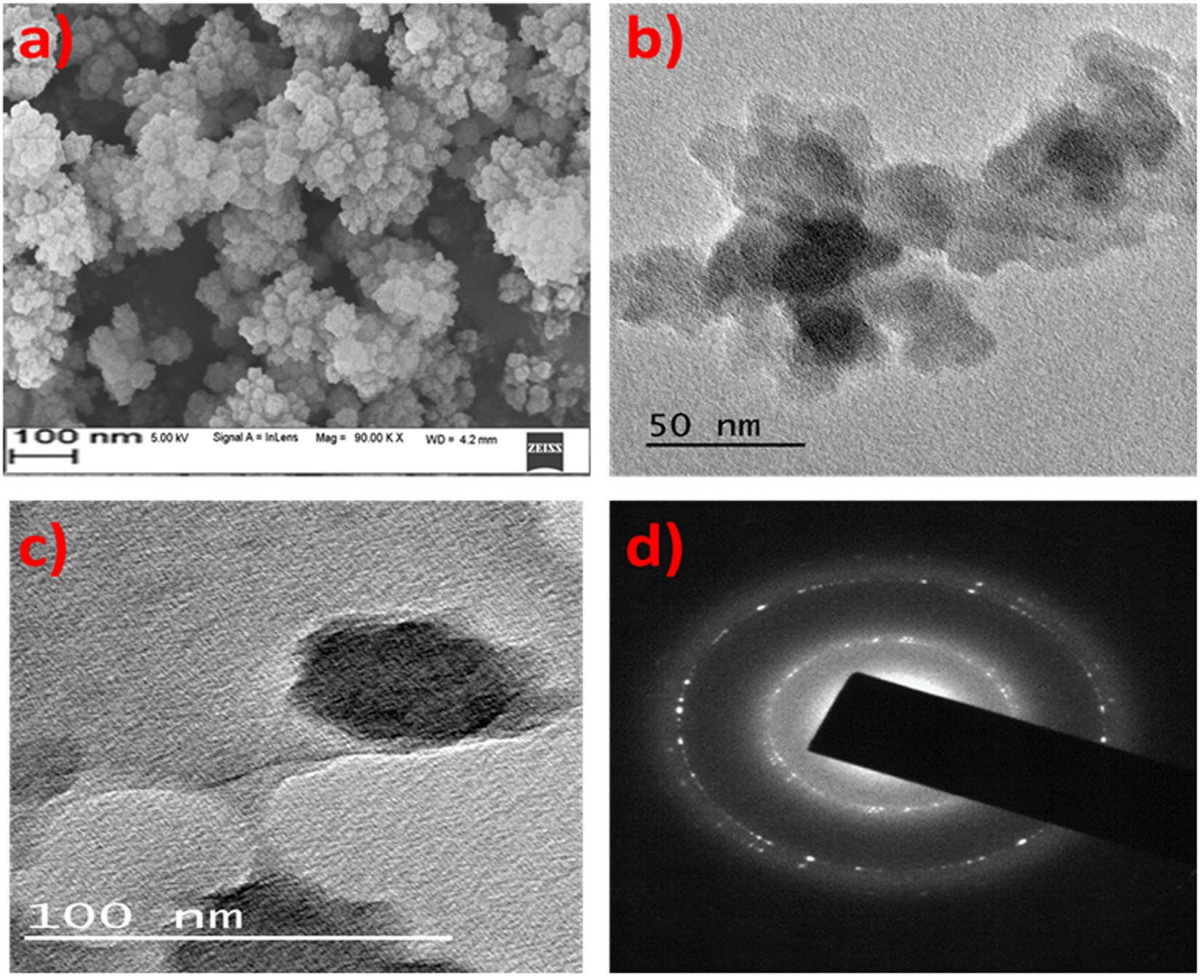
(**a**) FESEM image, (**b**,**c**), TEM images and (**d**) SAED pattern.

**Figure 5:**
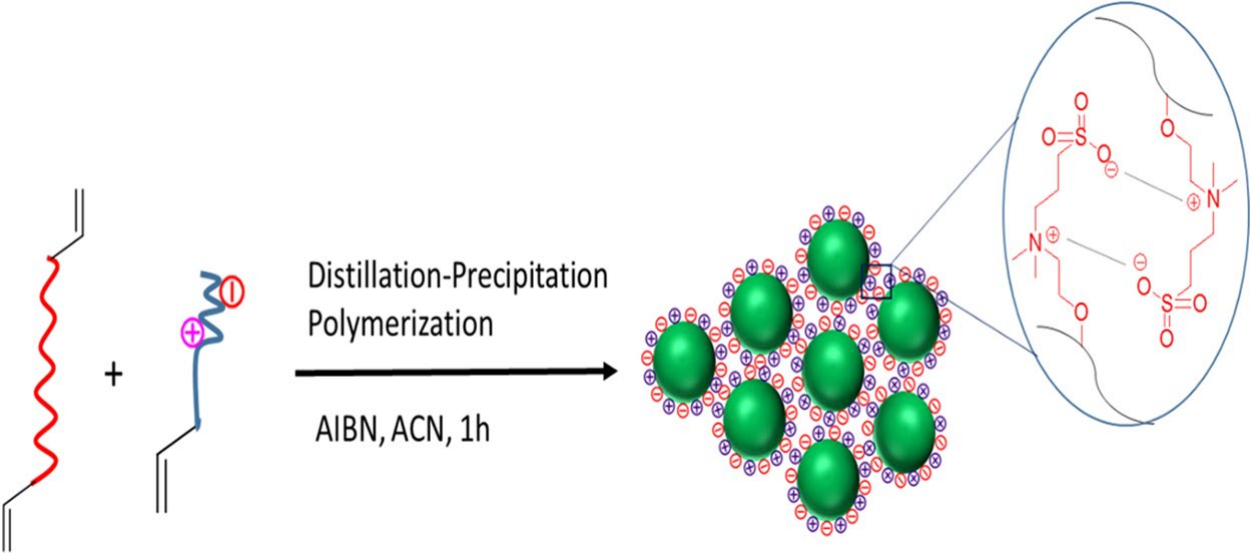
The schematic representation of nanoparticles synthesis and mechanism of agglomeration.

**Figure 6:**
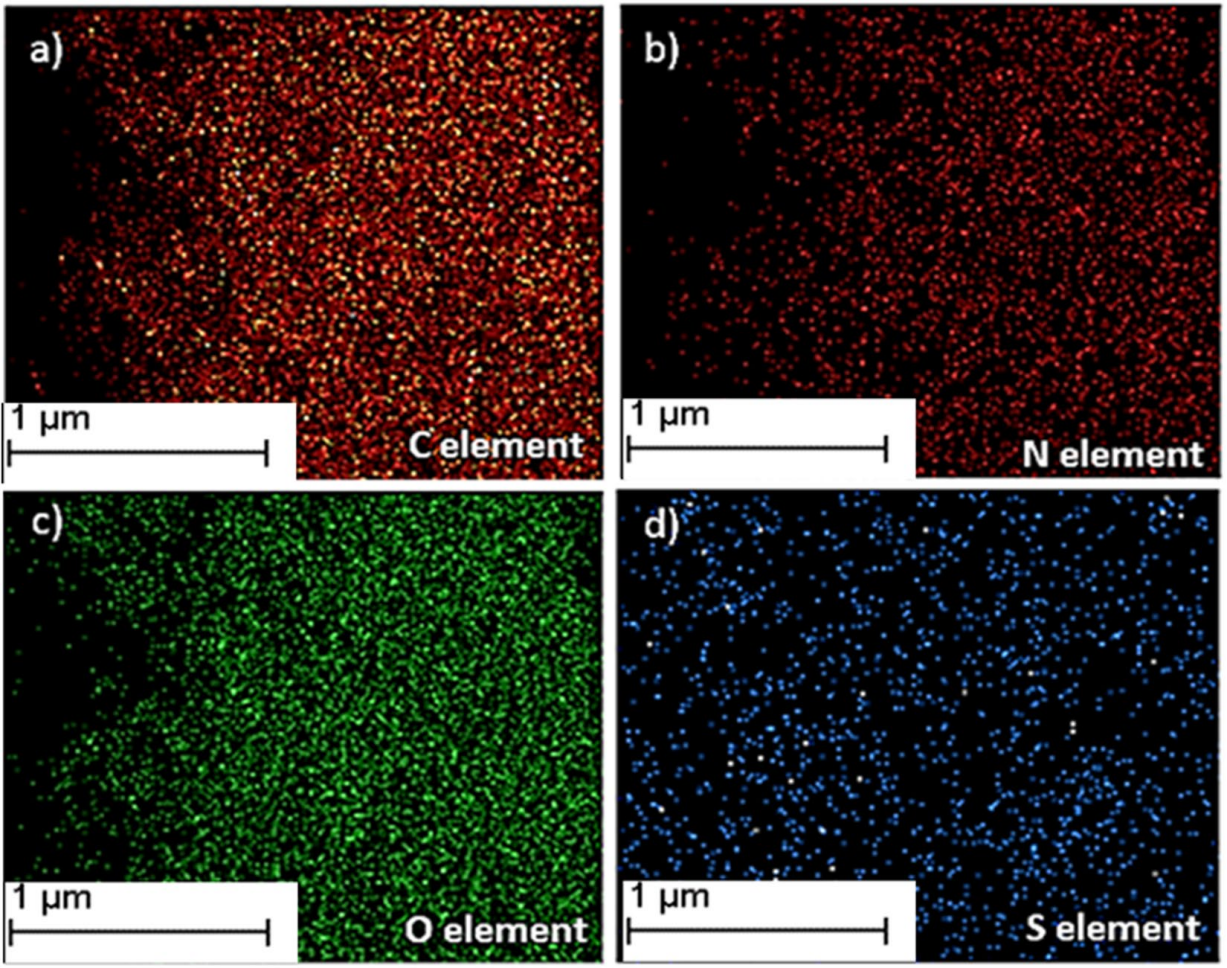
Element mapping of poly(MBAAm-co-SBMA) (**a–d**).

FT-IR spectra in Figure 3 represents the functional groups present in the poly(MBAAm-co-SBMA) and MBAAm. The peaks at 1656 cm^−1^ and 1529 cm^−1^ designate the stretching vibration of amide C = O and bending vibration of NH-CO. The peaks at 1722 cm^−1^and 1043 cm^−1^ indicate the ester (C = O) and sulfonate (S = O) stretching vibrations^62,63^. The peak at 1626 cm^−1^ attributed to the alkene C = C stretching vibration of MBAAm, which was not observed in the poly(MBAAm-co-SBMA) due to the polymerization reaction. This change is one of the reliable confirmation that the reaction had been completed. The peak at 3271 cm^−1^ indicates the stretching vibration of N-H in amide group of poly(MBAAm-co-SBMA). The peak at 1229 cm^−1^ due to C-N stretching vibration of the amide group. However, the peak at 954 cm^−1^ owing to the presence of C-N stretching vibration of quaternary ammonium group^63–65^. The above results indicated that the poly(MBAAm-co-SBMA) was comprised by the monomer SBMA and cross-linker MBAAm. The morphology of the as-prepared poly(MBAAm-co-SBMA) nanoparticles was visualized using field emission scanning electron microscope (FESEM) and transmission electron microscopy (TEM). As depicted in Figure 4, the nanoparticles exhibited comparatively physical uniform shape and size, with a diameter in the region of around less than 60 nm. Kaiguang *et al*. reported that in DPP the size of the nanoparticles does not depend on the amount of solvent removed, whereas the reaction temperature^54^, AIBN, and monomer concentration^51^ directly affects the size of the nanoparticles. Figure 4d presents the SAED pattern of the nanoparticles. It is clear from the picture that, the as-prepared nanoparticles exhibits small spots creating up a ring. It indicates that the nanoparticles are polycrystalline in nature^66^. In addition, the nanoparticles show slight agglomeration, which is clear from the TEM picture. The possible justification for such agglomeration may be due to the electrostatic attraction between the counterions. Figure 5 depicts the schematic representation of nanoparticles synthesis and possible mechanism of agglomeration. The similar kind of observation had been reported elsewhere^67,68^. Further, the elemental mapping analysis was carried out to confirm the presence of all the elements. Figure 6a–d show the distribution of C, N, O and S elements on poly(MBAAm-co-SBMA), among them S is the characteristic element of SBMA monomer. Consequently, Figure 6d confirms the presence of SBMA and uniform distribution.

#### Surface properties and hydrodynamic diameter

**Figure 7:**
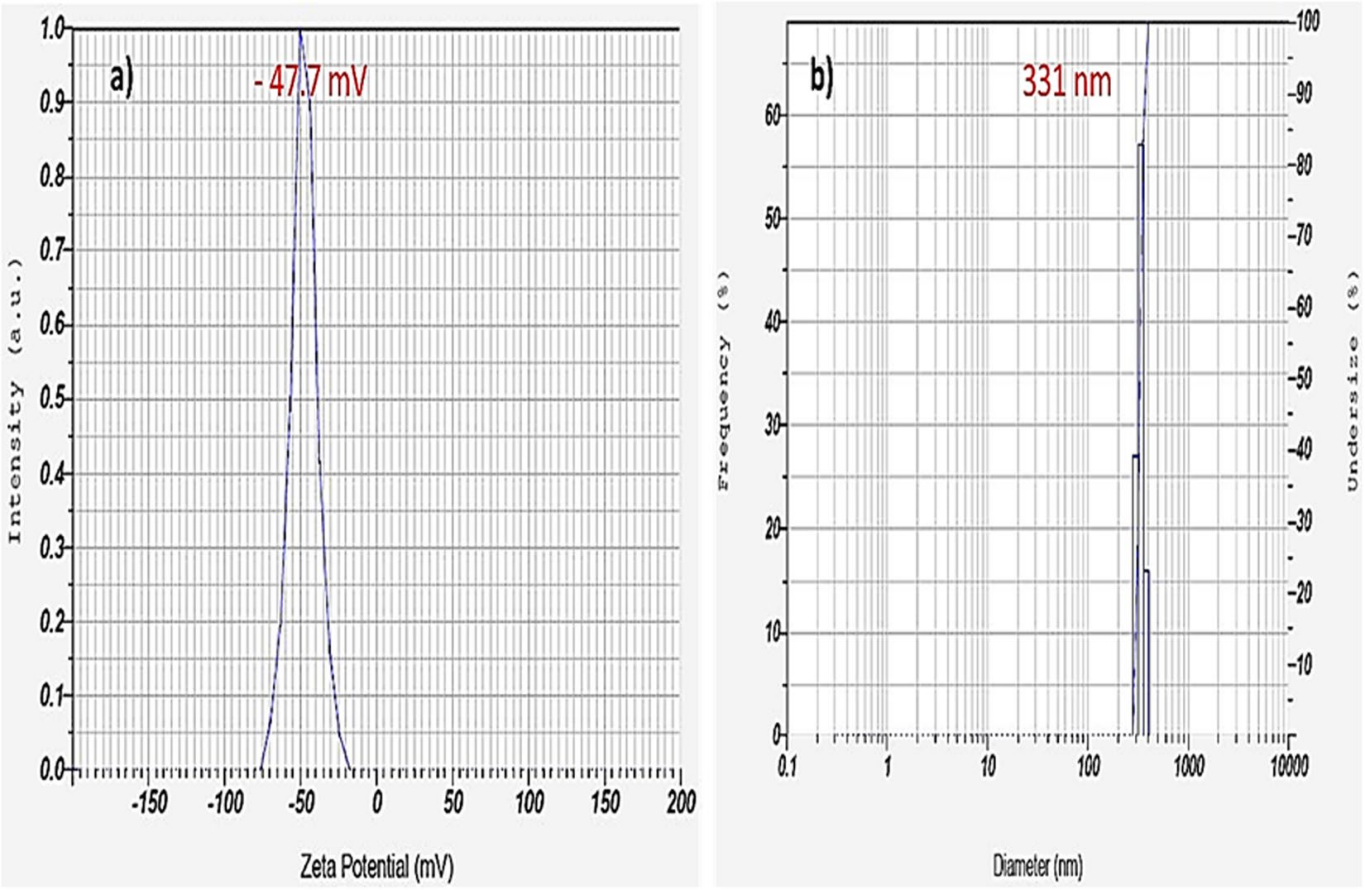
(**a)** ζ-potential and (**b**) hydrodynamic diameter of nanoparticles.

The surface charge of the synthesized poly(MBAAm-co-SBMA) nanoparticles was measured. As shown in Figure 7a, the nanoparticles exhibited the zeta (ζ) potential of − 47.7 mV. The negative ζ-potential of the nanoparticles could be attributed to the presence of sulfonate group. The pK_a_ value of sulfonate group is 2^69^ and the pK_b_ value of quaternary ammonium group is 5^70^. Consequently, the quaternary ammonium group signifies weaker base than the sulfonate group as acid. Therefore, the overall surface charges of the as-synthesized nanoparticles exhibit a negative charge in aqueous solution. According to Dorian *et al*. the dispersibility of the nanomaterial could be enhanced by coating with the carboxylic acid group, which provides negative ζ-potential to the material. As a result, the nanoparticle maintains the suspension over the extensive range of pH deprived of any agglomeration^71^. Similarly, the synthesized nanoparticles exhibit the negative ζ-potential and develop an electrical double layer, which avoids the nanoparticles from aggregating and preserves the stable dispersion in a variety of solvents through electrostatic repulsion. Further, the nanoparticles exhibited the BET surface area of 89.2 m^2^/g and mean pore diameter of 37 nm with the pore volume of 0.12 cc/g. In addition, the hydrodynamic diameter of the nanoparticle was 331 nm, which is presented in Figure 7b. The increase in the size was due to the slight aggregation of nanoparticles in water. Russell *et al*. reported that sulfonate group has strong tendency to form hydrogen bonding^72^. Therefore, aggregate formation was attributed to the formation of hydrogen bonding between the sulfonate group and water.

#### Thermal stability study

**Figure 8:**
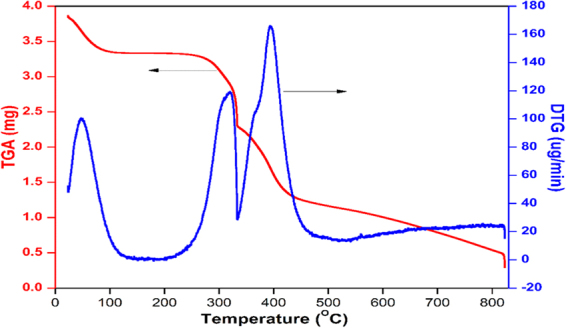
TGA and DTG curves of poly(MBAAm-co-SBMA).

The thermal stability of the nanoparticles was analyzed using TGA. The TGA analysis showed that the synthesized nanoparticle is thermally stable, as the onset of degradation is above 250 °C. Moreover, the curve contains three-stage degradation. The first weight loss between 25 and 105 °C due to the adsorbed water. The second weight loss from ca. 255 to 333 °C attributed to the degradation of a quaternary ammonium group. The third stage degradation in the region of ca. 340 to 450 °C ascribed to the removal of more stable oxygen functionalities. Figure 8 shows the TGA curves of the nanoparticles, along with its differential thermogravimetry (DTG) curve.

#### XRD analysis

**Figure 9:**
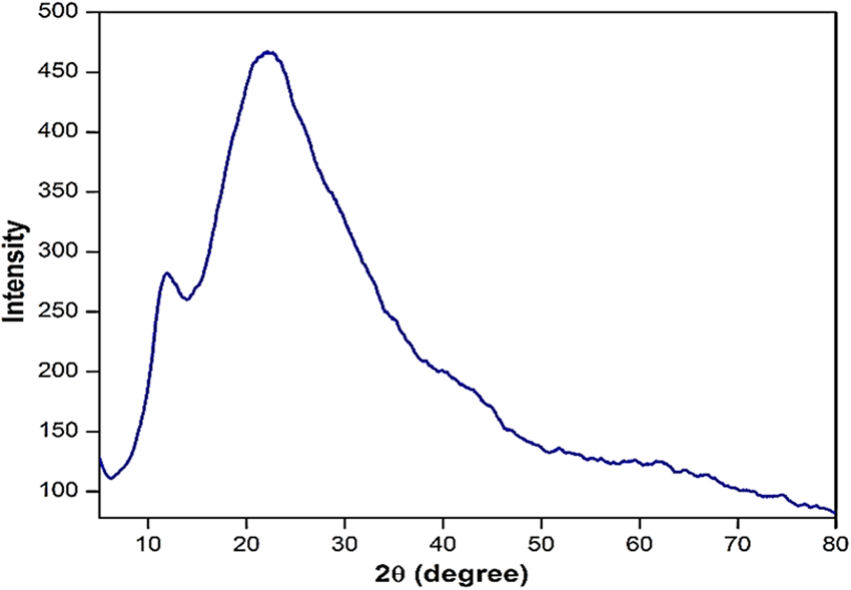
XRD pattern of poly(MBAAm-co-SBMA).

Figure 9 depicts the XRD pattern of the poly(MBAAm-co-SBMA) nanoparticles. Two intense broad bands centered at 2θ of ~11.88° and ~22.68° demonstrates that these nanoparticles are polycrystalline in nature. This result is well aligned with SAED pattern of the nanoparticles.

### Characterization of membranes

#### Surface hydrophilicity

The hydrophilicity of the as-prepared membranes was evaluated by measuring the contact angle and water uptake capacity. In general, it is believed that lower the contact angle higher will be the hydrophilicity^73,74^. Since the pristine membrane (M-0) is less hydrophilic in nature, it exhibited the higher contact angle of 80.0°. However for the nanocomposite membranes such as M-1, M-2, M-3 and M-4, the contact angle was observed 76.1°, 71.4°, 67.0° and 65.2° respectively (Table 3). The decrease in contact angle was attributed to the incorporation of hydrophilic poly(MBAAm-co-SBMA) nanoparticles. The hydrophilic functional group such sulfonate and amide group present in the nanoparticles was changed the interfacial free energy of the membrane. In addition, the sulfonic acid group has a greater water uptake capacity, which increases the surface hydrophilicity of the membrane.

**Table 3:** Membrane properties.

Membrane	Contact angle (°)	Water uptake (%)	Porosity (%)
M-0	80.0	29.5	41.6
M-1	76.1	48.2	53.1
M-2	71.4	53.1	61.4
M-3	67.0	61.7	66.5
M-4	65.2	56.4	62.2

#### Membrane Morphology

**Figure 10:**
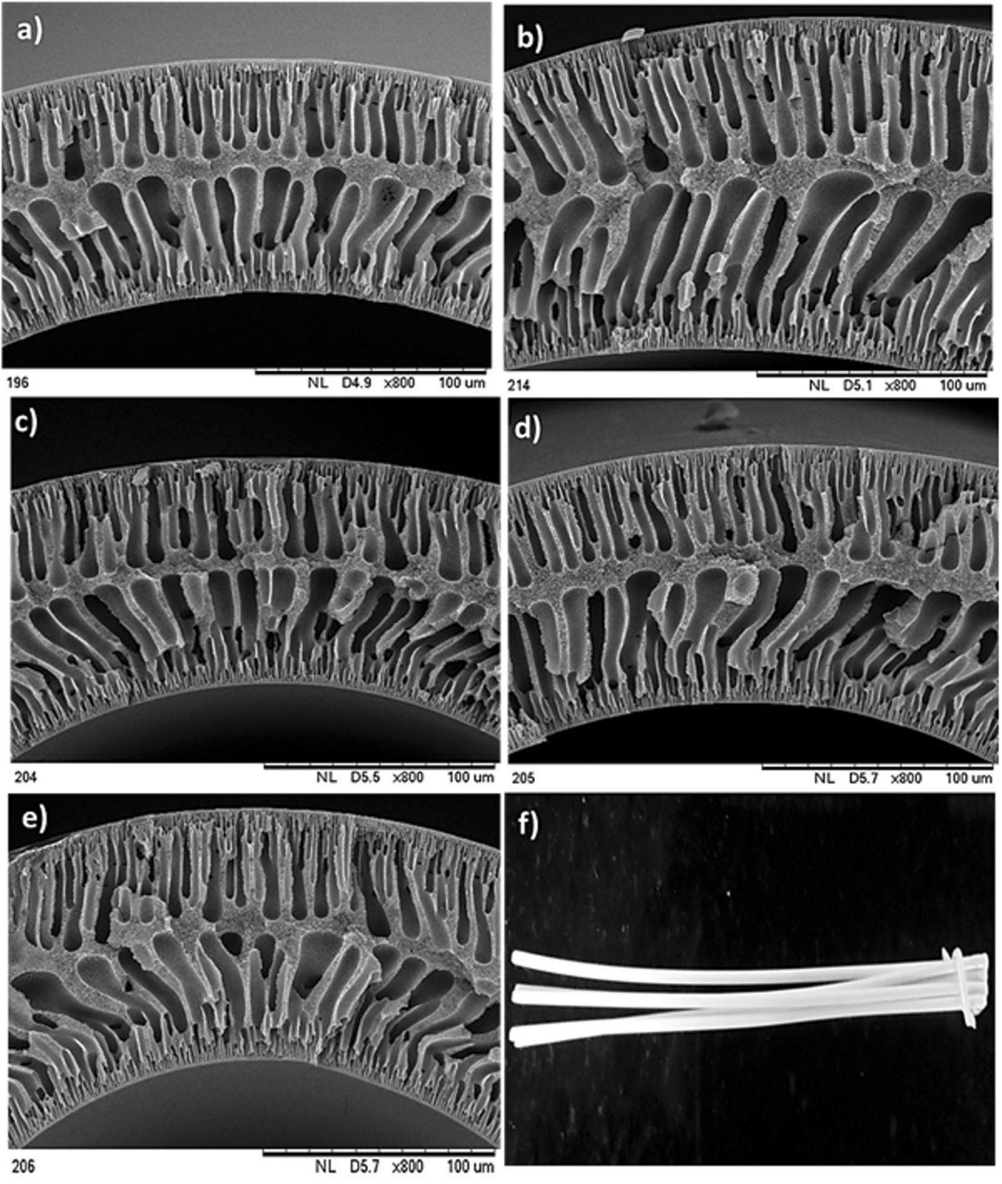
Cross-sectional SEM images of (**a**) M-0, (**b**) M-1, (**c**) M-2, (**d**) M-3 and (**e**) M-4 membranes magnified at 800X and digital photographic image of (**f**) M-3 membrane.

**Figure 11:**
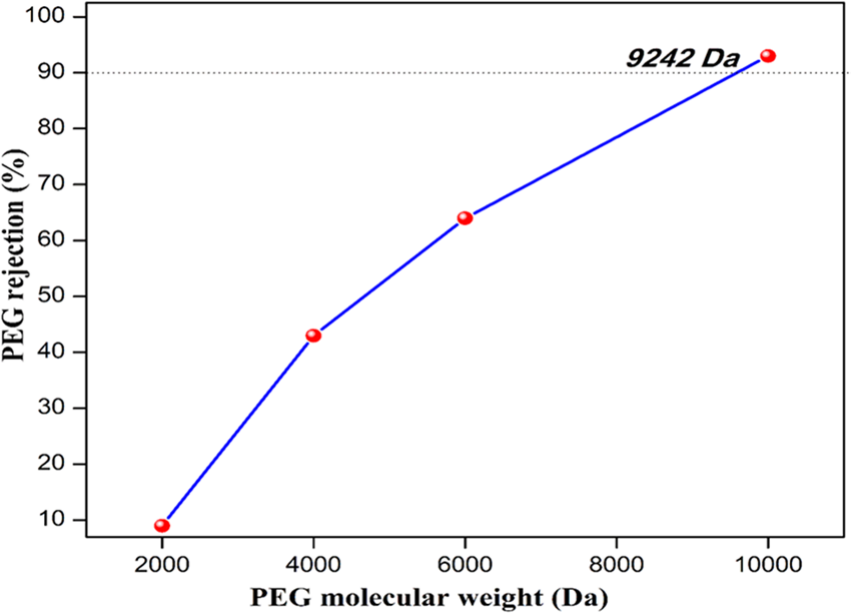
The MWCO curve of M-3 membrane.

The change in the morphology of the membranes upon the addition of nanoparticles was characterized using SEM. As shown in Figure  10, the nanocomposite hollow fiber membranes exhibit asymmetric structure with top skin layer, sub-layer, and fingerlike macrovoids. The sub-layer is sandwiched between the top and bottom fingerlike layer. As stated by McKelvey *et al*. the growth of macrovoids depends on the change in diffusion rate between non-solvent and solvent during phase inversion^75^. Since the pore-forming agent such as PVP was added to all the membranes invariably, the change in fingerlike projection between the prepared membranes was not observed distinctly upon the addition of nanoparticles. In addition, the air gap 1 cm was maintained throughout the spinning process to increase the flux. Subsequently, the phase inversion occurred on both outer and inner side of the membranes at a nearly concurrent rate and led to the formation of two layers of the finger-like structure. The reported results are consistent with the literature^76^. The normal digital photographic image of the HF membrane is depicted in Figure 10F. Besides, the MWCO of M-3 membrane is 9242 Da (Figure 11), which suggests that the as-prepared membrane is UF membrane^77^.

#### XPS analysis

**Figure 12:**
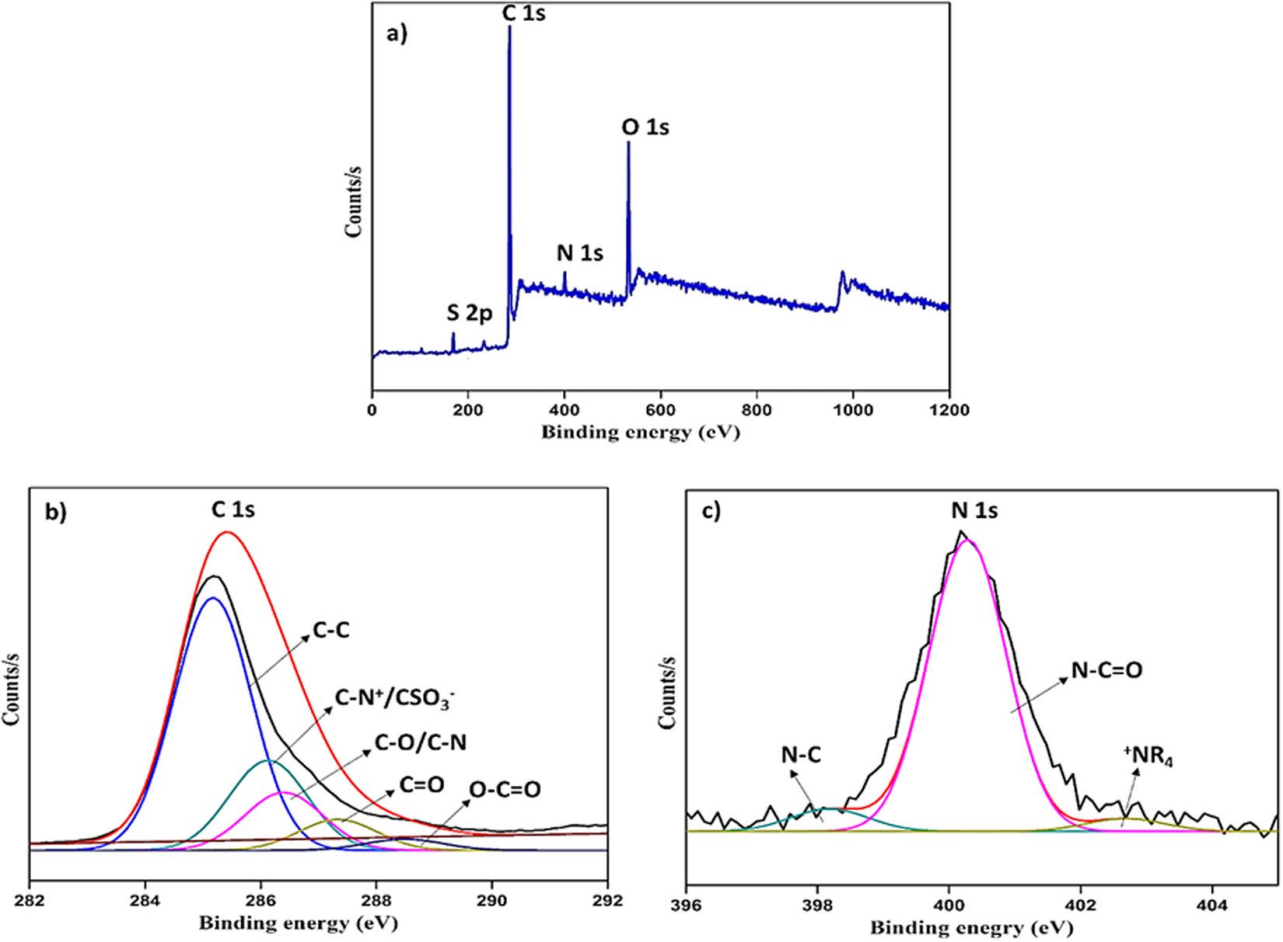
XPS spectra of M-3 membrane.

The M-3 membrane surface was analyzed by XPS and depicted in Figure 12. As shown in Figure 12a, the peaks at 168.38 eV, 285.18 eV, 400.18 eV and 532.18 eV were attributed to S 2p, C 1 s, N 1 s and O 1 s elements. Additionally, the deconvoluted peaks of C 1 s and N 1 s are presented in Figure 12b and c. In Figure 12b, the peaks at 285.21 eV, 286.14 eV, 286.36 eV, 287.11 eV and 288.15 eV were corresponding to C-C, C = O, C-N^+^/C-SO_3_^−^ and O-C = O. For N 1 s, N-C = O, N-C, and ^+^NR_4_ were observed at 400.28 eV, 398.18 eV, and 402.68 eV. The elemental composition (atomic %) of the nanocomposite HF membrane was observed as 77.35%, 15.81%, 2.76% and 4.08% for C, O, S and N elements respectively. Thereby, the existence of the nanoparticles in the membrane matrix was confirmed.

#### The surface charge of the membrane

**Figure 13:**
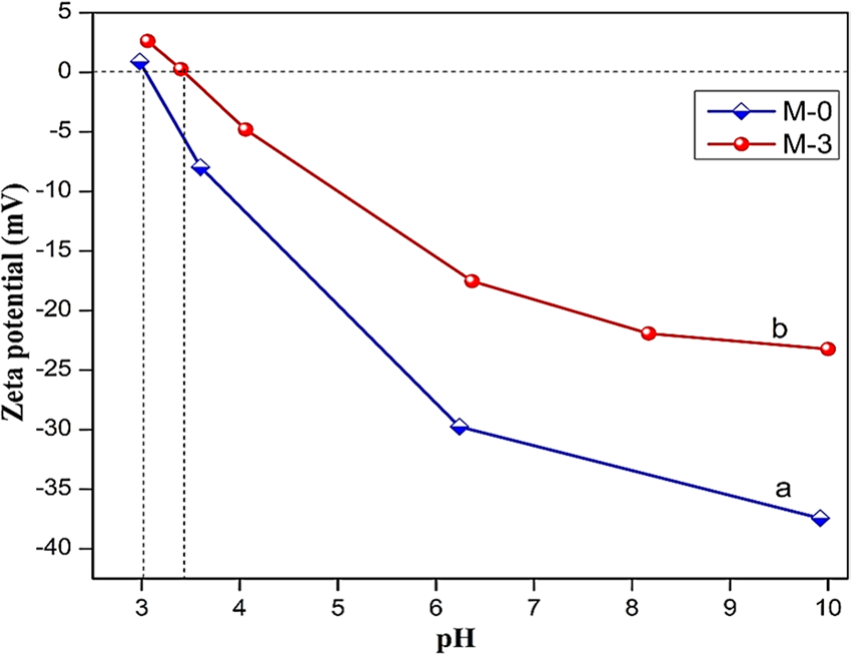
Zeta potential of (**a**) M-0 and (**b**) M-3 membranes.

As shown in Figure 13, the membranes M-0 and M-3 exhibited the negative charge over the entire pH range 4–10, and the absolute ζ-potential value was decreased to acidic pH values. The isoelectric point (IEP) of PSF neat (M-0) membrane was observed at pH 3.0, which is similar to the literature^78^. However, the IEP of M-3 membrane was detected at pH 3.4. The change in the IEP could be attributed to the incorporation of nanoparticles. In addition, the incorporated zwitterionic nanoparticles are negatively charged at pH 6.5. However, the ζ- potential of M-3 was less at pH 7 when compared to M-0 membrane. The reduced ζ- potential could be attributed to the intervention of cation adsorption from background electrolyte (KCl) on the surface, which decreases the negative charge density of the sulfonate group. As a result, the ζ- potential of the nanoparticle becomes less negative, that directly reduces the net charge of the membrane surface. Overall, the as-prepared membrane could exhibit negative charge over the large range of pH.

#### Permeability and antifouling performances

**Figure 14:**
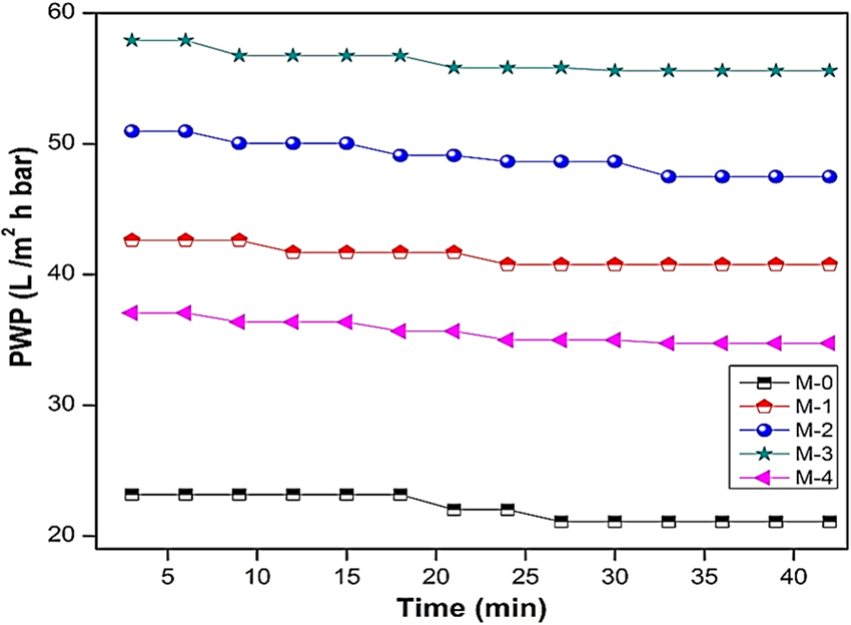
PWP of M-0, M-1, M-2, M-3 and M-4 membranes.

**Figure 15:**
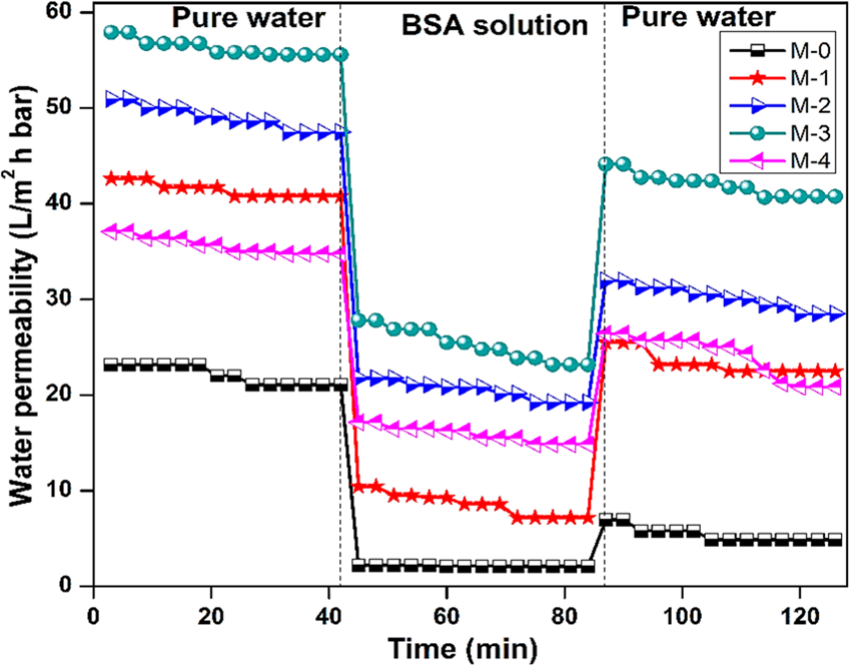
Time depended PWP in different conditions.

**Figure 16:**
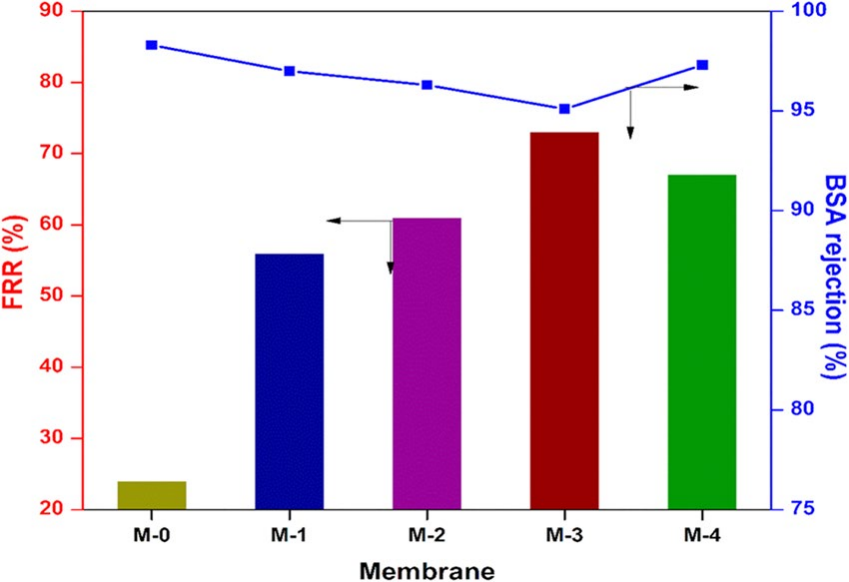
FRR and BSA rejection of membranes.

The permeation of water through the membrane is determined primarily by the surface hydrophilicity and pore size^79^. In order to evaluate the effect of the different poly(MBAAm-co-SBMA) nanoparticles content on the filtration performance of the as-made membranes, the pure water permeability (PWP) of all the UF membranes was measured; the results are presented in Figure 14. As shown, the PWP of the membranes increases with the enhancement of the concentration of nanoparticles. The pristine (M-0) exhibited the lowest PWP of 22 L/ m^2^ h bar. The membrane M-3, embedded with 0.5 wt% of nanoparticles exhibited the PWP of 56 L/ m^2^ h bar. A plausible explanation is that the added nanoparticles could be increased the surface hydrophilicity of the membranes. The increased hydrophilicity would enhance the rate of demixing during phase inversion. Further, the non-solvent inflow and solvent outflow would be more. Consequently, the porosity showed the increasing trend. As shown in Table 3, the porosity and water uptake increase as the concentration of nanoparticles increases. The membrane M-3 showed the highest porosity of 66.5% and water uptake of 61.7% compared to the pristine membrane M-0 of 41.6 and 29.5%. However, for the membrane M-4 with 1 wt% of nanoparticles, the porosity and water uptake reduced to 62.2 and 56.4%. The similar trend had been observed in PWP of M-4 membrane. The PWP was reduced to 35 L/ m^2^ h bar. The convincible reason for the reduction in porosity, water uptake, and PWP is that the embedded nanoparticles may lead to partial agglomeration, consequently blocking the pores of the membranes and increase the resistance towards the water permeation^46,80^. Figure 15 represents the time-dependent water permeability of the membranes at different conditions. The initial decline in the permeability of the water was due to the mechanical deformation of the membrane matrix^81^. In addition, Figure 15 indicates the increase of water permeability as the concentration of nanoparticle increases. However, during the BSA filtration, there was a sudden decline in the water permeability. The sudden decline was attributed to the adsorption of BSA molecules on the membrane surface, which blocks the polymeric membrane pores. The antifouling capacity of the as-made membranes was measured in terms of flux recovery ratio (FRR) and it is depicted in Figure 16. The membrane M-3 exhibited the FRR of 73% compared to the pristine membrane M-0 of 24%. The increased FRR of the M-3 was due to the increased hydrophilicity. It has been accepted widely that membrane surface decorated with zwitterionic substances can bestow outstanding antifouling ability^82–85^. Further, it forms the hydration layer over the membrane surface, which avoids the adsorption of foulants on the membrane surface. Moreover, the prepared nanocomposite membranes are exhibiting negative charge at the neutral pH. As a result, the BSA molecules are poorly adsorbed via electrostatic repulsion as the BSA molecules are negatively charged at pH 7.4. However, the membrane M-4 exhibited the reduced FRR of 67%. The reduced FRR was owing to the agglomeration of the nanoparticles, which encourages the adsorption of the foulant. In summary, the membrane with 0.5% of nanoparticle loading is the optimal concentration for the preparation of membrane.

#### Dye removal study

**Figure 17:**
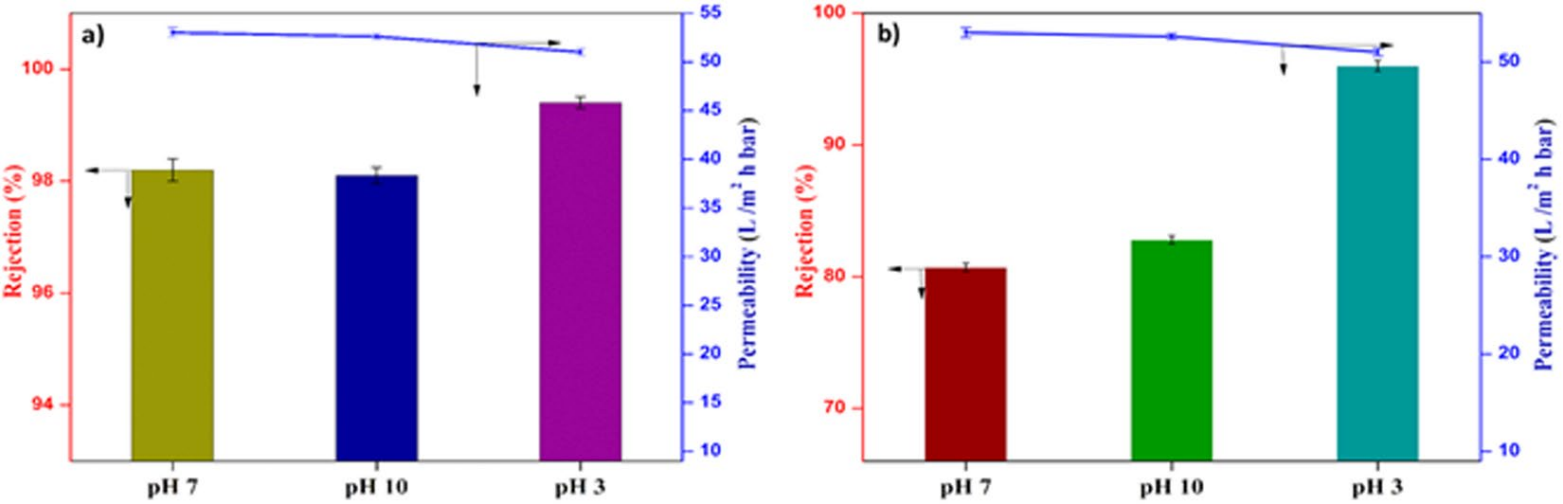
Permeability and rejection of (**a**) RB 5 and (**b**) RO 16 dyes at different pH.

**Figure 18:**
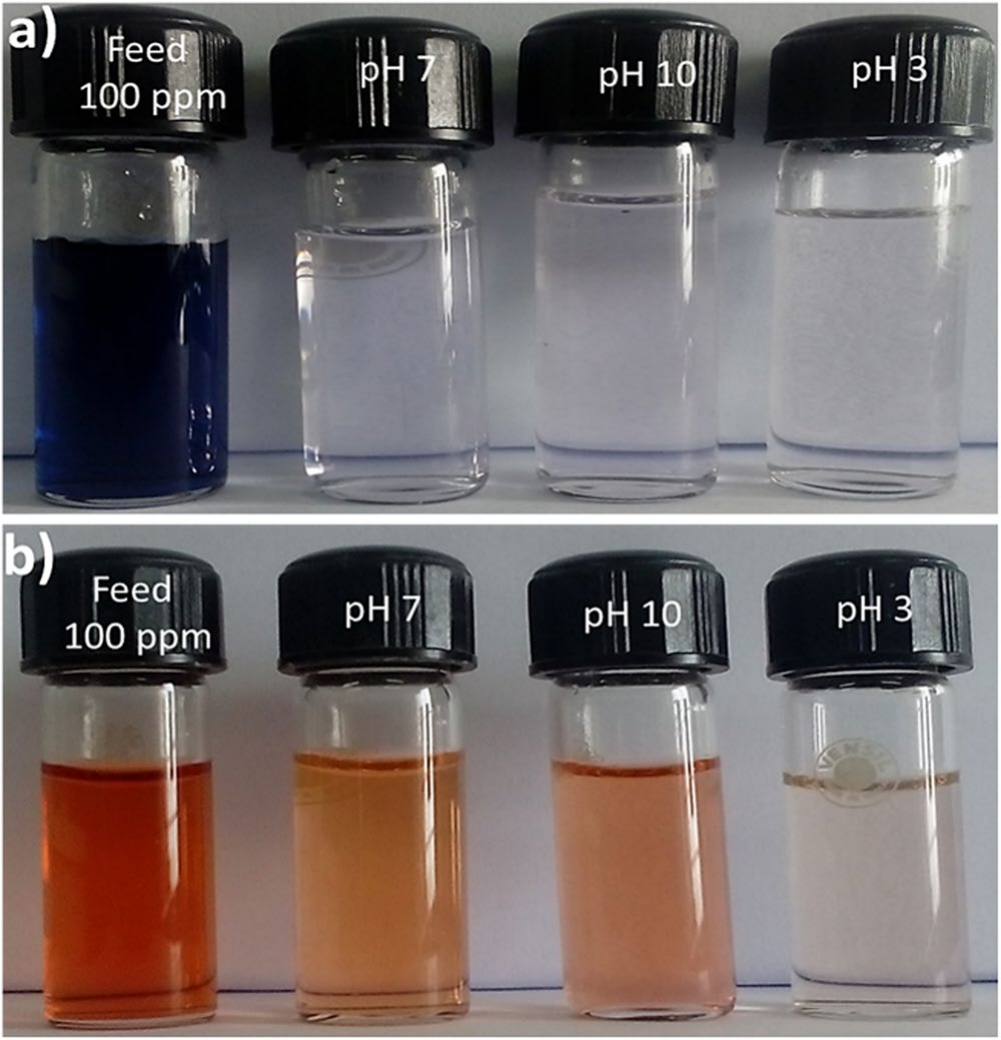
Digital photographs of (**a**) RB 5, (**b**) RO 16 feed and permeate at different pH.

The filtration ability of the M-3 membrane for the different dye solutions as a function of pH is depicted in Figure 17. In the pH range of 3–10, the membrane performance varies with the solution pH. As shown in Figure 17, in acidic pH the permeability of the dye decreases and rejection increases. In general, the sodium salt of dye molecules is highly soluble in water. However, while decreasing the pH to highly acidic side, the sulfonate groups present in the dye molecules are getting protonated and become a sulfonic acid group. As a result, the solubility and polarity of the dye molecules are decreased. Thus, the dye molecules are precipitated and aggregated largely at pH 3. In summary, the increased rejection owing to the aggregation of dye molecules and declined permeability due to the precipitation of dye, which is in good agreement with the reported literature^77^. At pH 10, the permeability of dye was reduced to a smaller extent. The reduced permeability could be due to the swelling of the membrane at the basic pH. The swelling could increase the thickness of the membrane^86^. Consequently, the permeability of the dye molecules was reduced to a smaller extent. Further, the rejection of RB 5 was high as compared to RO 16 at pH 7. The reason for the enhanced rejection was due to size exclusion mechanism i.e., the higher molecular weight of the former compared to later. In conclusion, the optimum pH for the removal of both the dye molecules is 7. The digital photographs of the feed and permeate of RB 5 and RO 16 are depicted in Fig. 18. The comparison of dye removal capacity of polymeric membranes from recent literature and the present study is illustrated in Table 4. Generally, the effluent from the textile and dyeing industry usually consist of dyes and salts^46^. In that respect, salts such as NaCl and Na_2_SO_4_rejection studies were carried out. The M-3 membrane exhibited the rejection in following order Na_2_SO_4_ (11%) > NaCl (7%), signifying that the nanocomposite membrane was negatively charged, which is consistent with the zeta potential result.

**Table 4:** Comparison of dye removal ability of polymeric membranes from recent literature and this study.

Membrane	Type of membrane	Dye	PWP (L/m^2^ h bar)^a^	DPF (L/m^2^ h bar)^b^	Dye rejection	Ref.
PES/GO-PSBMA	Loose NF	Reactive black 5	11.98	9.4	99.2	46
PAEK-COOH	Tight UF	Congo red	29.5	25	99.0	77
Sepro NF 6	Loose NF	Direct red 80	13.7	13.2	99.95	87
UH004 (Hydrophilic PES)	Tight UF	Direct red 80	27	26	99.9	88
Sepro NF 2 A	Loose NF	Direct red 80	10.5	9.6	99.98	87
PSf-poly(MBAAm-co-SBMA)	UF	Reactive black 5/ Reactive orange 16	56	51/51.8	98/80.7	This study

^a^PWP, pure water permeability.

^b^DPF, dye permeate flux.

## Conclusions

The zwitterionic polymer nanoparticles were synthesized via distillation-precipitation polymerization. The as-synthesized nanoparticles exhibited high surface area (89.2 m^2^/g), thermal and colloidal stability. The synthesized nanoparticles were successfully incorporated into polysulfone membrane matrix and the membranes were prepared by dry/wet phase inversion method. The M-3 nanocomposite membrane showed high pure water permeability of 56 L/ m^2^ h bar, rejection of reactive black 5 (>98%) and reactive orange 16 (>80.7%) with the dye permeability of 51 L/ m^2^ h bar and 51.8 L/ m^2^ h bar at dye concentration of 100 ppm, which has the molecular weight cut-off of 9242 Da. These results clearly reveal that the as-prepared membrane can be an attractive candidate for the treatment of industrial and textile wastewater treatment.

## References

[1] Shannon MA (2008). Science and technology for water purification in the coming decades. Nature.

[2] Wang Y, Chen J, Lu L, Lin Z (2013). Reversible switch between bulk MgCO3· 3H2O and Mg (OH) 2 micro/nanorods induces continuous selective preconcentration of anionic dyes. ACS Appl. Mater. Interfaces.

[3] Chethana M, Sorokhaibam LG, Bhandari VM, Raja S, Ranade VV (2016). Green Approach to Dye Wastewater Treatment Using Biocoagulants. ACS Sustain. Chem. Eng..

[4] Liu F, Chung S, Oh G, Seo TS (2012). Three-dimensional graphene oxide nanostructure for fast and efficient water-soluble dye removal. ACS Appl. Mater. Interfaces.

[5] Jiraratananon R, Sungpet A, Luangsowan P (2000). Performance evaluation of nanofiltration membranes for treatment of effluents containing reactive dye and salt. Desalination.

[6] Baughman GL, Weber EJ (1994). Transformation of dyes and related compounds in anoxic sediment: kinetics and products. ‎Environ. Sci. Technol..

[7] Weber EJ, Adams RL (1995). Chemical-and sediment-mediated reduction of the azo dye disperse blue 79. ‎Environ. Sci. Technol..

[8] Al-Degs YS, El-Barghouthi MI, El-Sheikh AH, Walker GM (2008). Effect of solution pH, ionic strength, and temperature on adsorption behavior of reactive dyes on activated carbon. Dyes Pigm..

[9] Lee J-W, Choi S-P, Thiruvenkatachari R, Shim W-G, Moon H (2006). Evaluation of the performance of adsorption and coagulation processes for the maximum removal of reactive dyes. Dyes Pigm..

[10] Ciardelli G, Ranieri N (2001). The treatment and reuse of wastewater in the textile industry by means of ozonation and electroflocculation. Water Res..

[11] Pala A, Tokat E (2002). Color removal from cotton textile industry wastewater in an activated sludge system with various additives. Water Res..

[12] Wang F (2015). Facile synthesis of a Ag(i)-doped coordination polymer with enhanced catalytic performance in the photodegradation of azo dyes in water. J. Mater. Chem. A.

[13] Yu S (2010). Impacts of membrane properties on reactive dye removal from dye/salt mixtures by asymmetric cellulose acetate and composite polyamide nanofiltration membranes. J. Membr. Sci..

[14] Chen G, Chai X, Po-Lock Y, Mi Y (1997). Treatment of textile desizing wastewater by pilot scale nanofiltration membrane separation. J. Membr. Sci..

[15] Marcucci M, Ciardelli G, Matteucci A, Ranieri L, Russo M (2002). Experimental campaigns on textile wastewater for reuse by means of different membrane processes. Desalination.

[16] Cassano A, Molinari R, Romano M, Drioli E (2001). Treatment of aqueous effluents of the leather industry by membrane processes: a review. J. Membr. Sci..

[17] Fersi C, Gzara L, Dhahbi M (2005). Treatment of textile effluents by membrane technologies. Desalination.

[18] Joshi M, Mukherjee A, Thakur B (2001). Development of a new styrene copolymer membrane for recycling of polyester fibre dyeing effluent. J. Membr. Sci..

[19] Baker, R. W. *Membrane technology*. (Wiley Online Library, 2000).

[20] Bes-Piá A (2005). Nanofiltration of textile industry wastewater using a physicochemical process as a pre-treatment. Desalination.

[21] Tang C, Chen V (2002). Nanofiltration of textile wastewater for water reuse. Desalination.

[22] Suksaroj C, Heran M, Allegre C, Persin F (2005). Treatment of textile plant effluent by nanofiltration and/or reverse osmosis for water reuse. Desalination.

[23] Shen L (2016). Low pressure UV-cured CS-PEO-PTEGDMA/PAN thin film nanofibrous composite nanofiltration membranes for anionic dye separation. J. Mater. Chem. A.

[24] Van der Bruggen B, Daems B, Wilms D, Vandecasteele C (2001). Mechanisms of retention and flux decline for the nanofiltration of dye baths from the textile industry. Sep. Purif. Technol..

[25] Van der Bruggen B, Cornelis G, Vandecasteele C, Devreese I (2005). Fouling of nanofiltration and ultrafiltration membranes applied for wastewater regeneration in the textile industry. Desalination.

[26] Al-Amoudi A, Lovitt RW (2007). Fouling strategies and the cleaning system of NF membranes and factors affecting cleaning efficiency. J. Membr. Sci..

[27] Mondal S, Ouni H, Dhahbi M, De S (2012). Kinetic modeling for dye removal using polyelectrolyte enhanced ultrafiltration. J. Hazard Mater..

[28] Rosberg R (1997). Ultrafiltration (new technology), a viable cost-saving pretreatment for reverse osmosis and nanofiltration—a new approach to reduce costs. Desalination.

[29] Hebbar, R. S., Isloor, A. M., Zulhairun, A., Abdullah, M. S. & Ismail, A. Efficient treatment of hazardous reactive dye effluents through antifouling polyetherimide hollow fiber membrane embedded with functionalized halloysite nanotubes. *J*. *Taiwan Inst*. *Chem*. *E* (2017).

[30] Ong YK (2014). Nanofiltration hollow fiber membranes for textile wastewater treatment: Lab-scale and pilot-scale studies. Chem. Eng. Sci..

[31] Mulder, J. *Basic principles of membrane technology*. (Springer Science & Business Media, 2012).

[32] Ho, W. W. & Sirkar, K. Membrane Handbook Van Nostrand-Reinhold. *New York* (1992).

[33] Ulbricht M, Riedel M, Marx U (1996). Novel photochemical surface functionalization of polysulfone ultrafiltration membranes for covalent immobilization of biomolecules. J. Membr. Sci..

[34] Jang J, Lee W (1994). Polyetherimide-modified high performance epoxy resin. Polymer journal.

[35] Nishimura S, Kohgo O, Kurita K, Kuzuhara H (1991). Chemospecific manipulations of a rigid polysaccharide: syntheses of novel chitosan derivatives with excellent solubility in common organic solvents by regioselective chemical modifications. Macromolecules.

[36] Brostow W, Hagg Lobland HE (2008). Predicting wear from mechanical properties of thermoplastic polymers. Polym. Eng. Sci..

[37] Quintanilla, V. A. Y. *Rejection of emerging organic contaminants by nanofiltration and reverse osmosis membranes: effects of fouling*, *modelling and water reus*e. (CRC Press/Balkema, 2010).

[38] Sun W, Liu J, Chu H, Dong B (2013). Pretreatment and membrane hydrophilic modification to reduce membrane fouling. Membranes.

[39] Mauter MS (2011). Antifouling ultrafiltration membranes via post-fabrication grafting of biocidal nanomaterials. ACS Applied Materials and Interfaces.

[40] Fan Z, Wang Z, Duan M, Wang J, Wang S (2008). Preparation and characterization of polyaniline/polysulfone nanocomposite ultrafiltration membrane. J. Membr. Sci..

[41] Schlenoff JB (2014). Zwitteration: coating surfaces with zwitterionic functionality to reduce nonspecific adsorption. Langmuir.

[42] Xiang T, Wang R, Zhao W-F, Sun S-D, Zhao C-S (2014). Covalent deposition of zwitterionic polymer and citric acid by click chemistry-enabled layer-by-layer assembly for improving the blood compatibility of polysulfone membrane. Langmuir.

[43] Yu H (2009). Enhancing antifouling property of polysulfone ultrafiltration membrane by grafting zwitterionic copolymer via UV-initiated polymerization. J. Membr. Sci..

[44] Shao Q, Jiang S (2015). Molecular understanding and design of zwitterionic materials. Adv. Mater..

[45] Jiang S, Cao Z (2010). Ultralow‐fouling, functionalizable, and hydrolyzable zwitterionic materials and their derivatives for biological applications. Adv. Mater..

[46] Zhu J (2016). Surface zwitterionic functionalized graphene oxide for a novel loose nanofiltration membrane. J. Mater. Chem. A.

[47] Xuan F, Liu J (2009). Preparation, characterization and application of zwitterionic polymers and membranes: current developments and perspective. Polym. Int..

[48] Zhao J (2016). Incorporating zwitterionic graphene oxides into sodium alginate membrane for efficient water/alcohol separation. ACS Appl. Mater. Interfaces.

[49] Ye G, Lee J, Perreault FO, Elimelech M (2015). Controlled architecture of dual-functional block copolymer brushes on thin-film composite membranes for integrated “defending” and “attacking” strategies against biofouling. ACS Appl. Mater. Interfaces.

[50] Liu T-Y (2015). Ion-responsive channels of zwitterion-carbon nanotube membrane for rapid water permeation and ultrahigh mono-/multivalent ion selectivity. ACS Nano.

[51] Bai F, Yang X, Huang W (2004). Synthesis of narrow or monodisperse poly (divinylbenzene) microspheres by distillation− precipitation polymerization. Macromolecules.

[52] Sosnowski S, Gadzinowski M, Slomkowski S (1996). Poly (l, l-lactide) microspheres by ring-opening polymerization. Macromolecules.

[53] Romack T, Maury E, DeSimone JM (1995). Precipitation polymerization of acrylic acid in supercritical carbon dioxide. Macromolecules.

[54] Yang K, Berg MM, Zhao C, Ye L (2009). One-pot synthesis of hydrophilic molecularly imprinted nanoparticles. Macromolecules.

[55] Liu J (2016). Synthesis of Zwitterionic Polymer Particles via Combined Distillation Precipitation Polymerization and Click Chemistry for Highly Efficient Enrichment of Glycopeptide. ACS Appl. Mater. Interfaces.

[56] Liu G, Yang X, Wang Y (2007). Preparation of monodisperse hydrophilic polymer microspheres with N, N′‐methylenediacrylamide as crosslinker by distillation precipitation polymerization. Polym. Int..

[57] Hebbar RS, Isloor AM, Ananda K, Abdullah MS, Ismail A (2017). Fabrication of a novel hollow fiber membrane decorated with functionalized Fe 2 O 3 nanoparticles: towards sustainable water treatment and biofouling control. New J. Chem..

[58] Pereira VR (2016). Preparation of polysulfone-based PANI–TiO 2 nanocomposite hollow fiber membranes for industrial dye rejection applications. RSc Adv..

[59] Kumar R, Ismail AF, Kassim MA, Isloor AM (2013). Modification of PSf/PIAM membrane for improved desalination applications using Chitosan coagulation media. Desalination.

[60] Tang YP (2016). Synthesis of hyperbranched polymers towards efficient boron reclamation via a hybrid ultrafiltration process. J. Membr. Sci..

[61] Shenvi S, Ismail A, Isloor AM (2014). Enhanced Permeation Performance of Cellulose Acetate Ultrafiltration Membranes by Incorporation of Sulfonated Poly (1, 4-phenylene ether ether sulfone) and Poly (styrene-co-maleic anhydride). Ind. Eng. Chem. Res..

[62] Moran PD (1995). Vibrational spectra of metal salts of bis(2-ethylhexyl)sulfosuccinate (AOT). J. Mater. Chem..

[63] Ji Y-L (2012). Novel composite nanofiltration membranes containing zwitterions with high permeate flux and improved anti-fouling performance. J. Membr. Sci..

[64] Ji Y, An Q, Zhao Q, Chen H, Gao C (2011). Preparation of novel positively charged copolymer membranes for nanofiltration. J. Membr. Sci..

[65] Shang H, Liu J, Zheng Y, Wang L (2009). Synthesis, characterization, and flocculation properties of poly (acrylamide‐methacryloxyethyltrimethyl ammonium chloride‐methacryloxypropyltrimethoxy silane). J. Appl. Polym. Sci..

[66] Egerton, R. F. *Physical principles of electron microscopy*. (Springer, 2005).

[67] Wang J-T, Wang L, Ji X, Liu L, Zhao H (2017). Synthesis of Zwitterionic Diblock Copolymers with Cleavable Biotin Groups at the Junction Points and Fabrication of Bioconjugates by Biotin–Streptavidin Coupling. Macromolecules.

[68] Chang Y, Shih YJ, Lai CJ, Kung HH, Jiang S (2013). Blood‐Inert Surfaces via Ion‐Pair Anchoring of Zwitterionic Copolymer Brushes in Human Whole Blood. Adv. Funct. Mater.

[69] Grainger, S. & El-Sayed, M. Biologically responsive hybrid biomaterials. *Artech House*, *Boston*, *MA*, *USA*, 171–190 (2010).

[70] Janson, J.-C. *Protein purification: principles*, *high resolution methods*, *and applications*. Vol. 151 (John Wiley & Sons, 2012).

[71] Hanaor D, Michelazzi M, Leonelli C, Sorrell CC (2012). The effects of carboxylic acids on the aqueous dispersion and electrophoretic deposition of ZrO 2. ‎J. Eur. Ceram. Soc..

[72] Russell VA, Evans CC, Li W, Ward MD (1997). Nanoporous molecular sandwiches: pillared two-dimensional hydrogen-bonded networks with adjustable porosity. Science.

[73] Hurwitz G, Guillen GR, Hoek EM (2010). Probing polyamide membrane surface charge, zeta potential, wettability, and hydrophilicity with contact angle measurements. J. Membr. Sci..

[74] Moideen K (2016). H.-K. Fabrication and characterization of new PSF/PPSU UF blend membrane for heavy metal rejection. Desalin. Water Treat..

[75] McKelvey SA, Koros WJ (1996). Phase separation, vitrification, and the manifestation of macrovoids in polymeric asymmetric membranes. J. Membr. Sci..

[76] Subramaniam M (2017). Hydrophilic hollow fiber PVDF ultrafiltration membrane incorporated with titanate nanotubes for decolourization of aerobically-treated palm oil mill effluent. Chem. Eng. J..

[77] Liu C, Mao H, Zheng J, Zhang S (2017). Tight ultrafiltration membrane: Preparation and characterization of thermally resistant carboxylated cardo poly (arylene ether ketone) s (PAEK-COOH) tight ultrafiltration membrane for dye removal. J. Membr. Sci..

[78] Kim K, Lee K, Cho K, Park C (2002). Surface modification of polysulfone ultrafiltration membrane by oxygen plasma treatment. J. Membr. Sci..

[79] Zhang G (2016). Ultralow Oil-Fouling Heterogeneous Poly (ether sulfone) Ultrafiltration Membrane via Blending with Novel Amphiphilic Fluorinated Gradient Copolymers. Langmuir.

[80] Hebbar RS, Isloor AM, Ananda K, Ismail A (2016). Fabrication of polydopamine functionalized halloysite nanotube/polyetherimide membranes for heavy metal removal. J. Mater. Chem. A.

[81] Reinsch VE, Greenberg AR, Kelley SS, Peterson R, Bond LJ (2000). A new technique for the simultaneous, real-time measurement of membrane compaction and performance during exposure to high-pressure gas. J. Membr. Sci..

[82] Hadidi M, Zydney AL (2014). Fouling behavior of zwitterionic membranes: Impact of electrostatic and hydrophobic interactions. J. Membr. Sci..

[83] Rohani MM, Zydney AL (2012). Protein transport through zwitterionic ultrafiltration membranes. J. Membr. Sci..

[84] He Y (2008). Molecular simulation studies of protein interactions with zwitterionic phosphorylcholine self-assembled monolayers in the presence of water. Langmuir.

[85] Mi L, Giarmarco MM, Shao Q, Jiang S (2012). Divalent cation-mediated polysaccharide interactions with zwitterionic surfaces. Biomaterials.

[86] Wang J (2016). Zwitterionic functionalized layered double hydroxides nanosheets for a novel charged mosaic membrane with high salt permeability. J. Membr. Sci..

[87] Lin J (2015). Fractionation of direct dyes and salts in aqueous solution using loose nanofiltration membranes. J. Membr. Sci..

[88] Lin J (2016). Tight ultrafiltration membranes for enhanced separation of dyes and Na 2 SO 4 during textile wastewater treatment. J. Membr. Sci..

